# Efficacy of an Eastern Healthy Dietary Pattern-modified ketogenic diet in patients with obesity and polyendocrine metabolic ovarian syndrome

**DOI:** 10.3389/fendo.2026.1948375

**Published:** 2026-09-28

**Authors:** Qiuxia Chen, Pianhong Zhang, Minhui Dai, Yuting Feng, Xiao Yu, Pingping Zhao, Xiaoming He

**Affiliations:** 1 The Second Affiliated Hospital of Zhejiang Chinese Medical University, Hangzhou, China; 2 The Second Affiliated Hospital of Zhejiang University, Hangzhou, China; 3 Xiangya Hospital of Central South University, Changsha, China; 4 Shulan International Medical College, Zhejiang Shuren University, Hangzhou, China

**Keywords:** Eastern Healthy Dietary Pattern, insulin resistance, ketogenic diet, obesity, polyendocrine metabolic ovarian syndrome, visceral adiposity

## Abstract

**Objective:**

To evaluate the efficacy and safety of an Eastern Healthy Dietary Pattern (EHDP)-modified ketogenic diet in Chinese patients with obesity and polyendocrine metabolic ovarian syndrome (PMOS; formerly polycystic ovary syndrome [PCOS]) and to provide evidence for a culturally adapted nutritional intervention.

**Methods:**

This single-arm, self-controlled study enrolled 55 patients with PMOS and obesity (body mass index [BMI] ≥28 kg/m²) from the Clinical Nutrition Clinic of the Second Affiliated Hospital of Zhejiang Chinese Medical University between February 2024 and September 2025. Participants underwent a 12-week phased dietary intervention. Body composition, metabolic indices, reproductive hormones, and clinical symptoms were assessed before and after the intervention.

**Results:**

During the intervention, blood β-hydroxybutyrate concentrations remained within the target range of 0.5-3.0 mmol/L and reached 1.24 ± 0.41 mmol/L at week 12, indicating good adherence. Mean body weight decreased by 9.97 kg (10.63%), body fat mass by 7.53 kg, and visceral fat area by 35.81 cm², whereas skeletal muscle mass decreased by 1.60 kg. The homeostasis model assessment of insulin resistance (HOMA-IR) decreased by 48.8%, and triglyceride concentrations decreased from 1.60 ± 0.91 to 1.08 ± 0.49 mmol/L. Alanine aminotransferase and aspartate aminotransferase concentrations also decreased significantly, whereas low-density lipoprotein cholesterol and small dense low-density lipoprotein cholesterol did not increase significantly. Total testosterone and luteinizing hormone concentrations decreased by 35.8% and 55.5%, respectively, and the luteinizing hormone/follicle-stimulating hormone ratio approached the normal range. Serum 25-hydroxyvitamin D increased from 17.86 ± 3.52 to 23.90 ± 4.12 ng/mL. Clinical symptoms improved, with no clinically significant adverse changes in the assessed safety parameters (all *P* > 0.05).

**Conclusion:**

A 12-week EHDP-modified ketogenic diet was associated with improvements in body composition, metabolic indices, reproductive hormones, and clinical symptoms in Chinese patients with PMOS and obesity, without clinically significant safety concerns. This culturally adapted approach may offer a practical nutritional strategy for this population; controlled trials are warranted to confirm these findings.

**Clinical trial registration:**

https://www.chictr.org.cn/showproj.html?proj=176830, identifier ChiCTR2200063473.

## 1 Introduction

Polyendocrine metabolic ovarian syndrome (PMOS), formerly known as polycystic ovary syndrome (PCOS), is one of the most common endocrine and metabolic disorders among women of reproductive age and is characterized by insulin resistance, hyperandrogenism, ovulatory dysfunction, and obesity (1). Recent epidemiologic data from China indicate an overall PMOS prevalence of 10.01%; approximately 80% of affected women have overweight or obesity (BMI ≥24 kg/m²), and 31.47% have obesity (BMI ≥28 kg/m²). Abdominal obesity (waist circumference >85 cm) is present in 48.25% of patients with PMOS, a substantially higher proportion than that observed in the general population (2, 3). Obesity and PMOS may reinforce one another: adipose tissue inflammation and reduced insulin sensitivity can aggravate insulin resistance and hyperandrogenism, whereas hyperandrogenism can promote adipose accumulation, particularly visceral adiposity. This cycle contributes to menstrual irregularity, infertility, and increased cardiovascular risk (4, 5). Nutritional management of PMOS with obesity should therefore address weight control, insulin resistance, and endocrine dysfunction.

Lifestyle and nutritional interventions are first-line treatments for PMOS. Conventional approaches generally emphasize total energy restriction, but poor long-term adherence and limited durability of metabolic improvements can reduce their effectiveness (6). By markedly restricting carbohydrate intake to approximately 5%-10% of total energy, a ketogenic diet induces nutritional ketosis and may promote weight loss, improve insulin sensitivity, and modulate reproductive hormones (7). Clinical studies have reported body-weight reductions of 8%-12%, improvements of more than 30% in HOMA-IR, and lower free testosterone concentrations within 12 weeks among patients with PMOS and obesity (8, 9). However, conventional ketogenic diets are often high in saturated fat and low in dietary fiber, which may increase the risk of dyslipidemia during prolonged use. They may also be difficult to reconcile with traditional Chinese dietary patterns, in which carbohydrate-rich staple foods are prominent, potentially limiting long-term adherence and wider clinical implementation (10). The concept of “flexible ketosis,” which uses gradual carbohydrate restriction to reduce intervention-related discomfort, has therefore been proposed (11). Mediterranean-style ketogenic diets similarly emphasize unsaturated fats and fiber-rich foods and may reduce adverse effects such as dyslipidemia and constipation while supporting gut microbial diversity and metabolic flexibility (12, 13). These approaches provide a useful basis for developing a culturally adapted dietary intervention for PMOS.

The Eastern Healthy Dietary Pattern (EHDP), exemplified by the Jiangnan diet, is an optimized model of the traditional Chinese diet described in the 2022 Chinese Dietary Guidelines. It emphasizes plant-based foods, high-quality protein sources such as fish, shrimp, and soy products, unsaturated fats such as olive and camellia oils, limited refined carbohydrates, reduced sodium, and minimally processed foods (14, 15). Previous studies suggest that the EHDP may reduce body fat percentage and visceral fat area and improve lipid profiles and chronic inflammation in individuals with obesity (16). These features align closely with the nutritional needs of patients with PMOS and obesity. However, clinical evidence on an EHDP combined with a ketogenic diet in this population remains limited.

Following a multistep international consensus process, the condition was renamed polyendocrine metabolic ovarian syndrome (PMOS) in 2026. The new term better reflects the syndrome’s multisystem endocrine-metabolic pathophysiology and avoids implying that pathological ovarian cysts are its defining feature (17).

Accordingly, we integrated the culturally relevant features of the EHDP with the metabolic principles of a ketogenic diet to develop a regimen characterized by low carbohydrate intake, adequate dietary fiber, high-quality fats, and plant-derived bioactive compounds. The regimen retains the carbohydrate restriction required to promote ketone production while addressing potential nutritional limitations of a conventional ketogenic diet through foods typical of the Jiangnan diet, including fish, shrimp, soy products, and dark-green vegetables. In this single-arm, self-controlled study, we evaluated the effects of this EHDP-modified ketogenic diet on body composition, metabolic indices, and reproductive endocrine function in patients with PMOS and obesity. The study was intended to provide preliminary evidence for a culturally adapted nutritional intervention for PMOS.

## 2 Materials and methods

### 2.1 Study participants

We enrolled 55 patients with PMOS and obesity who attended the Clinical Nutrition Clinic of the Second Affiliated Hospital of Zhejiang Chinese Medical University between February 2024 and September 2025. The study was approved by the Ethics Committee of the Second Affiliated Hospital of Zhejiang Chinese Medical University (approval no. 2023-066-R01), and all participants provided written informed consent before enrollment.

No formal *a priori* sample-size or power calculation was performed for this exploratory, single-arm study. The analysis included all 55 participants who enrolled and completed the 12-week intervention; consequently, the estimates should be interpreted as preliminary, and the study may have limited power to detect smaller effects.

The inclusion criteria were as follows: (1) PMOS diagnosed according to the Rotterdam criteria specified in the Chinese Guidelines for the Diagnosis and Treatment of Polycystic Ovary Syndrome (2018), which were published under the former PCOS terminology; (2) age 18–45 years; (3) BMI ≥28 kg/m², consistent with the Chinese diagnostic criterion for obesity; and (4) willingness to participate, provision of written informed consent, and ability to complete the intervention and all study assessments.

The exclusion criteria were as follows: (1) severe cardiac, hepatic, renal, or thyroid dysfunction; diabetes mellitus; malignant disease; or autoimmune disease; (2) use of weight-loss medication, hormone therapy, or another weight-loss intervention within the previous 3 months; (3) allergy to foods included in the ketogenic diet, dietary restrictions, or inability to adhere to the prescribed regimen; and (4) a history of alcohol dependence, psychiatric illness, or cognitive impairment.

### 2.2 Dietary intervention

#### 2.2.1 Core dietary principles

All participants underwent a 12-week ketogenic dietary intervention adapted according to the principles of the EHDP. The intervention was implemented in progressive phases, and registered clinical dietitians provided individualized dietary counseling and adherence support throughout the study.

The dietary regimen combined low carbohydrate intake, high-quality fats, moderate protein intake, and adequate dietary fiber with the core features of the EHDP. The specific requirements were as follows:

1. Carbohydrates: Daily intake was limited to 30–50 g, primarily from low-glycemic-index foods such as dark-green leafy vegetables and small amounts of root vegetables.
2. Fats: Fat provided 60%-70% of total daily energy. Unsaturated fat sources, including camellia, olive, and flaxseed oils, were prioritized, together with moderate amounts of nuts. Trans fats and excessive saturated fat were avoided.
3. Protein: Protein provided 20%-27% of total daily energy. Preferred sources included fish and shrimp (300–500 g/week), soy products such as tofu and soy milk (50–100 g/day), eggs (1-2/day), and lean meat.
4. Dietary fiber: The target intake was ≥25 g/day, supplied by fresh vegetables (200–250 g/day, mainly dark-green leafy vegetables), edible mushrooms, and konjac. A 10–15 g/day fiber supplement was provided when dietary intake was insufficient.
5. Additional recommendations: Daily salt intake was limited to <5 g, and daily fluid intake was 2000–2500 mL. Alcohol, sugar-sweetened beverages, and processed foods were prohibited.

The reference menu provided 1200–1400 kcal/day. An example daily menu included stir-fried eggs with whitebait and spinach (250 g) and sweet corn (50 g) for breakfast; stir-fried Chinese cabbage with lean meat and tofu puffs (275 g) for lunch; and poached shrimp with stir-fried leaf lettuce (350 g) for dinner. Camellia oil (70 g) and salt (5 g) were allocated across the day.

#### 2.2.2 Intervention phases and adherence monitoring

The intervention comprised three progressive phases, with adherence monitored throughout:

1. Baseline period (1 week before the intervention): Clinical dietitians assessed habitual intake using a 24-hour dietary recall and a food frequency questionnaire and developed an individualized dietary plan and menu for each participant.
2. Adaptation period (weeks 1-2; flexible induction of ketosis): Carbohydrate intake was gradually reduced from 100 g/day to the target range of 30–50 g/day to minimize gastrointestinal symptoms and the risk of hypoglycemia. Participants received weekly follow-up by telephone or in person to address dietary questions and practical difficulties.
3. Intensification period (weeks 3-12): Participants followed the target dietary regimen, with in-person follow-up every 4 weeks to assess adherence and adjust the regimen when necessary.
4. Adherence assessment: Participants recorded food types, quantities, and cooking methods in a daily food diary. Adherence was assessed weekly using the food diary and urine ketone test strips (target, + to +++). For participants with adherence <80%, the dietary plan was adjusted and additional counseling was provided.

### 2.3 Outcome measures and assessment methods

All outcomes were collected and measured according to standardized procedures. Body composition, routine biochemical indices, and clinical symptoms were assessed at baseline (week 0) and at weeks 4, 8, and 12. Reproductive hormones and ultrasonographic liver parameters were assessed at baseline and week 12. Participants fasted for 12 hours before each assessment and were instructed to avoid strenuous exercise, alcohol, and high-fat foods during the preceding 24 hours.

#### 2.3.1 Body composition

Body weight, BMI, percentage body fat (PBF), visceral fat area (VFA), skeletal muscle mass (SMM), fat-free mass (FFM), soft lean mass (SLM), segmental fat mass, and total body water (TBW) were measured using a multifrequency bioelectrical impedance analyzer (InBody 770; Biospace, Seoul, Republic of Korea). Participants wore light clothing, fasted, and emptied their bladders before measurement. All measurements were obtained according to the manufacturer’s instructions.

#### 2.3.2 Biochemical and hormonal assessments

Routine biochemical measurements included blood urea nitrogen (BUN), creatinine (Cr), uric acid (UA), fasting plasma glucose (FPG), alanine aminotransferase (ALT), aspartate aminotransferase (AST), total protein (TP), albumin (ALB), total bilirubin (TBIL), direct bilirubin (DBIL), indirect bilirubin (IBIL), gamma-glutamyl transferase (GGT), triglycerides (TG), total cholesterol (TC), high-density lipoprotein cholesterol (HDL-C), low-density lipoprotein cholesterol (LDL-C), and small dense LDL cholesterol (sd-LDL). Measurements were performed using an automated biochemical analyzer (Hitachi 7600; Hitachi, Tokyo, Japan).

Fasting insulin (FINS) was measured by chemiluminescence immunoassay, glycated hemoglobin (HbA1c) by high-performance liquid chromatography, and β-hydroxybutyrate (β-OHB) by an enzymatic assay. Two-hour postchallenge plasma glucose (2hPG) was measured enzymatically after a 75-g oral glucose load. HOMA-IR was calculated as follows: HOMA-IR = (FPG × FINS)/22.5.

Follicle-stimulating hormone (FSH), luteinizing hormone (LH), estradiol (E2), progesterone (PRO), total testosterone (T), prolactin (PRL), and anti-Müllerian hormone (AMH) were measured using a chemiluminescence immunoassay system (cobas 8000; Roche Diagnostics, Basel, Switzerland). The LH/FSH ratio was then calculated.

Serum 25-hydroxyvitamin D [25(OH)D] was measured by liquid chromatography-tandem mass spectrometry.

#### 2.3.3 Liver ultrasonography and clinical symptom assessment

Liver stiffness and the liver attenuation coefficient were measured using an ultrasound-based liver fibrosis assessment system (STZH-LESCAN-1; China). Measurements were obtained through the seventh to ninth intercostal spaces between the right anterior and midaxillary lines. The mean of three valid measurements was used for analysis.

Menstrual symptoms were assessed as follows:

1. Menstrual cycle and duration: Participants recorded the first day and duration of each menstrual period in a symptom diary. Cycle length was defined as the interval between the first days of two consecutive menstrual periods.
2. Dysmenorrhea: Severity was evaluated using the Clinical Symptom Rating Scale for Menstrual Disorders (range, 0-3): 0, no pain; 1, occasional mild pain without interference with daily activities; 2, frequent moderate pain requiring simple measures such as heat therapy and causing mild interference with daily activities; and 3, recurrent severe pain requiring medication and markedly limiting daily activities or requiring bed rest.
3. Menstrual blood loss: Blood loss was estimated from sanitary pad saturation (80% saturation ≈10 mL) and scored from 0 to 3: 0, <80 mL (normal or slightly reduced); 1, 80–100 mL (mildly increased); 2, 100–120 mL (moderately increased); and 3, >120 mL (markedly increased, often accompanied by anemia).

### 2.4 Statistical analysis

Statistical analyses were performed using SPSS version 26.0 (IBM Corp., Armonk, NY, USA), and figures were generated using OriginPro 2021 (OriginLab Corp., Northampton, MA, USA). All tests were two-sided, and *P* < 0.05 was considered statistically significant. The normality of continuous variables was assessed using the Shapiro-Wilk test. For outcomes measured at baseline and week 12, normally distributed continuous variables were presented as mean ± standard deviation and compared using paired t tests; non-normally distributed variables were presented as median (interquartile range) [M (Q1, Q3)] and compared using Wilcoxon signed-rank tests. For continuous outcomes measured at baseline and weeks 4, 8, and 12, one-way repeated-measures analysis of variance was used when model assumptions were met, with Greenhouse-Geisser correction when sphericity was violated and Bonferroni-adjusted pairwise comparisons. Non-normally distributed or ordinal repeated outcomes, including menstrual symptom scores, were analyzed using the Friedman test followed by Bonferroni-adjusted Wilcoxon signed-rank comparisons. All 55 participants completed follow-up; therefore, no imputation for missing data was required.

## 3 Results

The study included 55 patients with PMOS and obesity, with a mean age of 27.8 ± 5.8 years. All participants completed the 12-week EHDP-modified ketogenic diet intervention, and no participants were lost to follow-up. Blood β-OHB concentrations remained within the target range of 0.5-3.0 mmol/L during the intervention and increased from 0.12 ± 0.05 mmol/L at baseline to 1.24 ± 0.41 mmol/L at week 12 (*P* < 0.001), indicating successful induction of nutritional ketosis and good dietary adherence.

### 3.1 Changes in body composition

After 12 weeks, body weight and fat-related measures had decreased significantly, whereas losses in muscle-related measures were smaller (**Table 1**). As shown in **Figure 1A**, body weight, PBF, VFA, and body fat mass (BFM) decreased throughout the intervention. The trajectories of BFM and VFA were similar, suggesting broadly parallel reductions in total and visceral adiposity. PBF and body weight also followed similar trajectories. **Figure 1B** shows significant reductions in regional BFM in the arms, legs, and trunk. By comparison, changes in total and regional FFM, SMM, and SLM were more modest. **Figure 1C** shows small decreases in TBW, intracellular water (ICW), and extracellular water (ECW) (all *P* < 0.05). The ECW/TBW ratio changed slightly but remained stable overall (*P* = 0.001).

**Table 1:** Changes in body composition from baseline to week 12 and paired t test results.

Variable	Unit	Baseline	Week 12	Mean difference	*P* value
Body weight	kg	83.77 ± 11.73	73.80 ± 11.15	-9.97	<0.001
Body mass index (BMI)	kg/m²	32.58 ± 3.59	28.74 ± 3.47	-3.84	<0.001
Fat-free mass index (FFMI)	kg/m²	18.18 ± 1.21	17.27 ± 1.24	-0.92	<0.001
Fat mass index (FMI)	kg/m²	14.40 ± 2.89	11.48 ± 2.76	-2.92	<0.001
Body fat mass (BFM)	kg	37.00 ± 8.11	29.48 ± 7.71	-7.53	<0.001
Soft lean mass (SLM)	kg	44.15 ± 4.84	41.78 ± 4.65	-2.37	0.013
Fat-free mass (FFM)	kg	46.76 ± 5.16	44.32 ± 4.96	-2.44	0.016
Skeletal muscle mass (SMM)	kg	25.82 ± 3.00	24.22 ± 2.88	-1.6	0.008
Percentage body fat (PBF)	%	44.00 ± 4.46	39.48 ± 5.12	-4.53	<0.001
InBody score		62.17 ± 6.61	66.67 ± 6.52	4.5	<0.001
Obesity degree		155.17 ± 17.14	136.85 ± 16.60	-18.33	<0.001
Right-arm FFM	kg	2.55 ± 0.36	2.31 ± 0.35	-0.24	0.002
Visceral fat area (VFA)	cm²	180.27 ± 36.17	144.46 ± 39.58	-35.81	<0.001
Left-arm FFM	kg	2.52 ± 0.36	2.28 ± 0.35	-0.25	0.001
Trunk FFM	kg	21.62 ± 2.27	20.06 ± 2.19	-1.56	0.001
Right-leg FFM	kg	7.20 ± 0.87	6.84 ± 0.89	-0.36	0.034
Left-leg FFM	kg	7.17 ± 0.87	6.80 ± 0.88	-0.37	0.023
Right-arm BFM	kg	3.28 ± 1.21	2.38 ± 0.99	-0.91	<0.001
Left-arm BFM	kg	3.32 ± 1.21	2.41 ± 1.00	-0.9	<0.001
Trunk BFM	kg	18.11 ± 3.43	14.63 ± 3.54	-3.48	<0.001
Right-leg BFM	kg	5.41 ± 1.13	4.38 ± 1.07	-1.02	<0.001
Left-leg BFM	kg	5.37 ± 1.12	4.36 ± 1.06	-1.01	<0.001
Total body water (TBW)	mL	34.32 ± 3.69	32.56 ± 3.63	-1.76	0.014
Intracellular water (ICW)	mL	21.29 ± 2.25	20.10 ± 2.21	-1.19	0.007
Extracellular water (ECW)	mL	13.03 ± 1.45	12.46 ± 1.43	-0.57	0.045
ECW/TBW ratio		0.38 ± 0.01	0.38 ± 0.00	0	<0.001

Data are presented as mean ± SD. BFM, body fat mass; ECW, extracellular water; FFM, fat-free mass; FFMI, fat-free mass index; FMI, fat mass index; ICW, intracellular water; PBF, percentage body fat; SLM, soft lean mass; SMM, skeletal muscle mass; TBW, total body water; VFA, visceral fat area.

**Figure 1:**
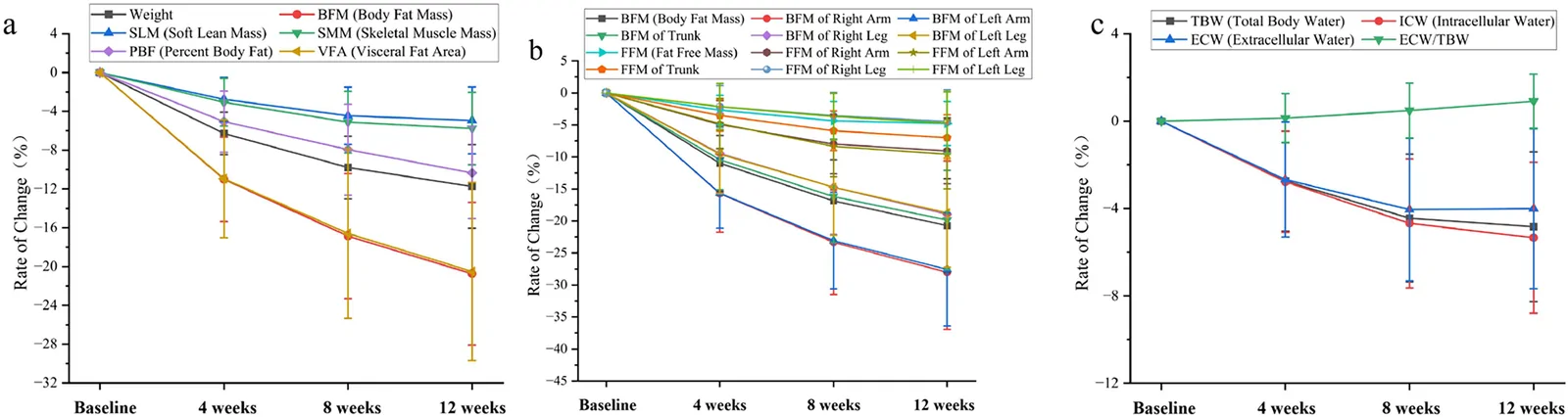
Changes in body composition during the intervention. **(A)** Changes in body weight and fat- and muscle-related measures. **(B)** Regional changes in body fat mass (BFM) and fat-free mass (FFM). **(C)** Changes in total body water (TBW), intracellular water (ICW), extracellular water (ECW), and the ECW/TBW ratio. *P* < 0.001 versus baseline.

As shown in **Table 1**, mean body weight decreased by 9.97 kg (10.63%), BMI by 3.84 kg/m², PBF by 4.53 percentage points, and VFA by 35.81 cm². BFM decreased significantly in the limbs and trunk. SMM decreased by 1.60 kg, whereas FFM and SLM decreased by 2.44 and 2.37 kg, respectively (all *P* < 0.05). The InBody score increased by 4.5 points (*P* < 0.001), and the obesity degree score decreased by 18.33 points (*P* < 0.001). These findings indicate that the reduction in fat mass exceeded the reduction in muscle-related measures.

### 3.2 Changes in metabolic indices

#### 3.2.1 Glucose metabolism and insulin resistance

Glucose metabolism and insulin resistance improved after the EHDP-modified ketogenic diet intervention (**Figure 2**). FPG decreased from 5.34 ± 0.65 to 4.94 ± 0.49 mmol/L, and FINS decreased from 24.61 ± 12.72 to 13.04 ± 7.93 pmol/L (both *P* < 0.001). HOMA-IR decreased by 48.8% (*P* < 0.001). HbA1c also decreased, from 5.72 ± 0.41% to 5.38 ± 0.35% (*P* < 0.001), indicating improved short- and longer-term glycemic indices.

**Figure 2:**
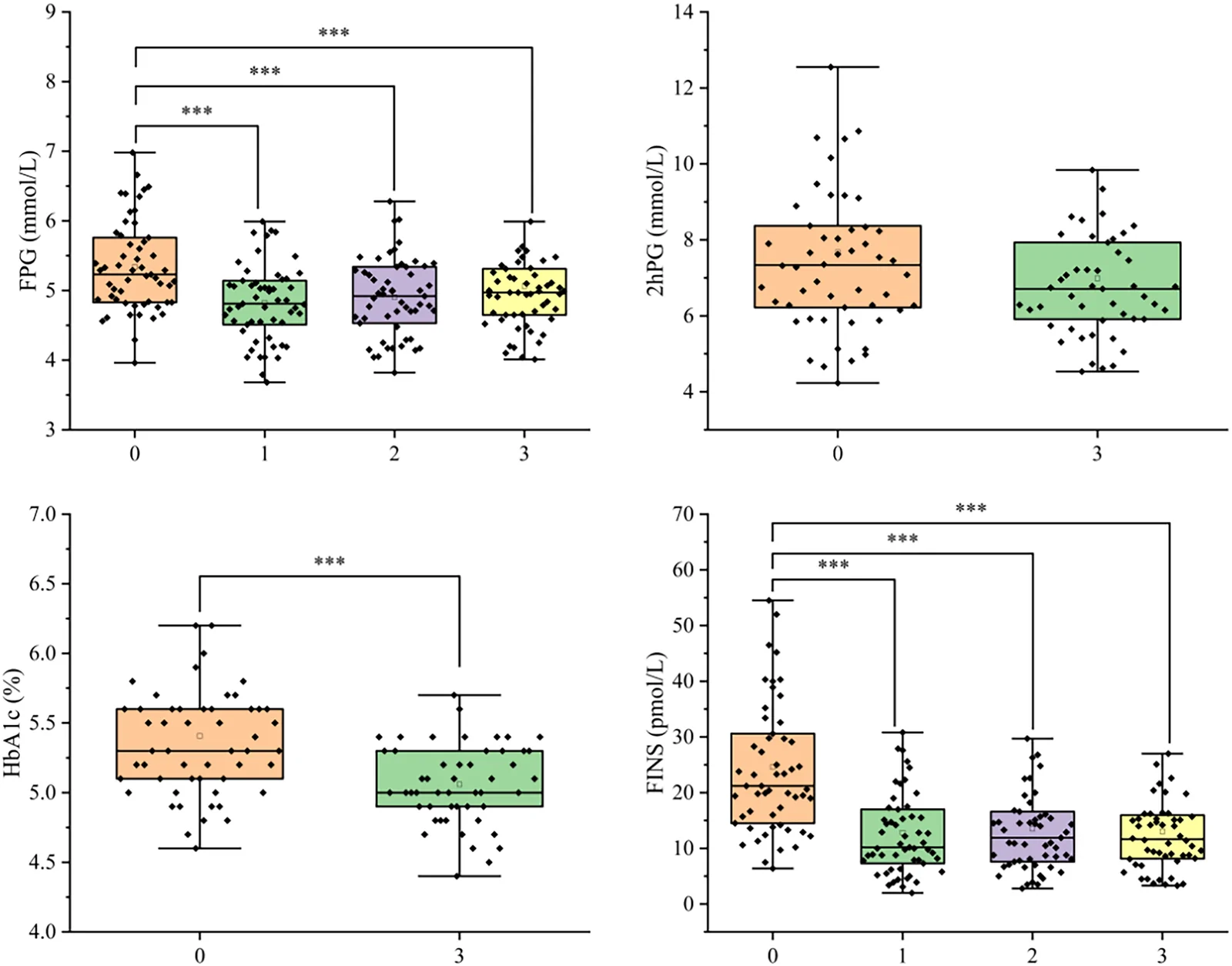
Changes in glycemic indices during the Eastern Healthy Dietary Pattern-modified ketogenic diet intervention. FPG, fasting plasma glucose; 2hPG, 2-hour postchallenge plasma glucose; HbA1c, glycated hemoglobin; FINS, fasting insulin; HOMA-IR, homeostasis model assessment of insulin resistance. The numeric x-axis codes denote assessment time points: 0, baseline (week 0); 1, week 4; 2, week 8; and 3, week 12. Panels displaying only 0 and 3 were assessed only at baseline and week 12. Brackets identify the compared time points. ***P < 0.001.

#### 3.2.2 Lipid profile and liver-related indices

The lipid profile improved after the intervention (**Figure 3**). TG decreased by 32.5%, from 1.60 ± 0.91 to 1.08 ± 0.49 mmol/L (*P* < 0.001). HDL-C decreased slightly early in the intervention and then increased to 1.25 ± 0.28 mmol/L at week 12, compared with 1.12 ± 0.24 mmol/L at baseline (*P* = 0.023). Despite fat providing 60%-70% of total energy, TC, LDL-C, and sd-LDL did not increase significantly (all *P* > 0.05). Thus, the intervention did not adversely affect the measured atherogenic lipid indices during the 12-week period.

**Figure 3:**
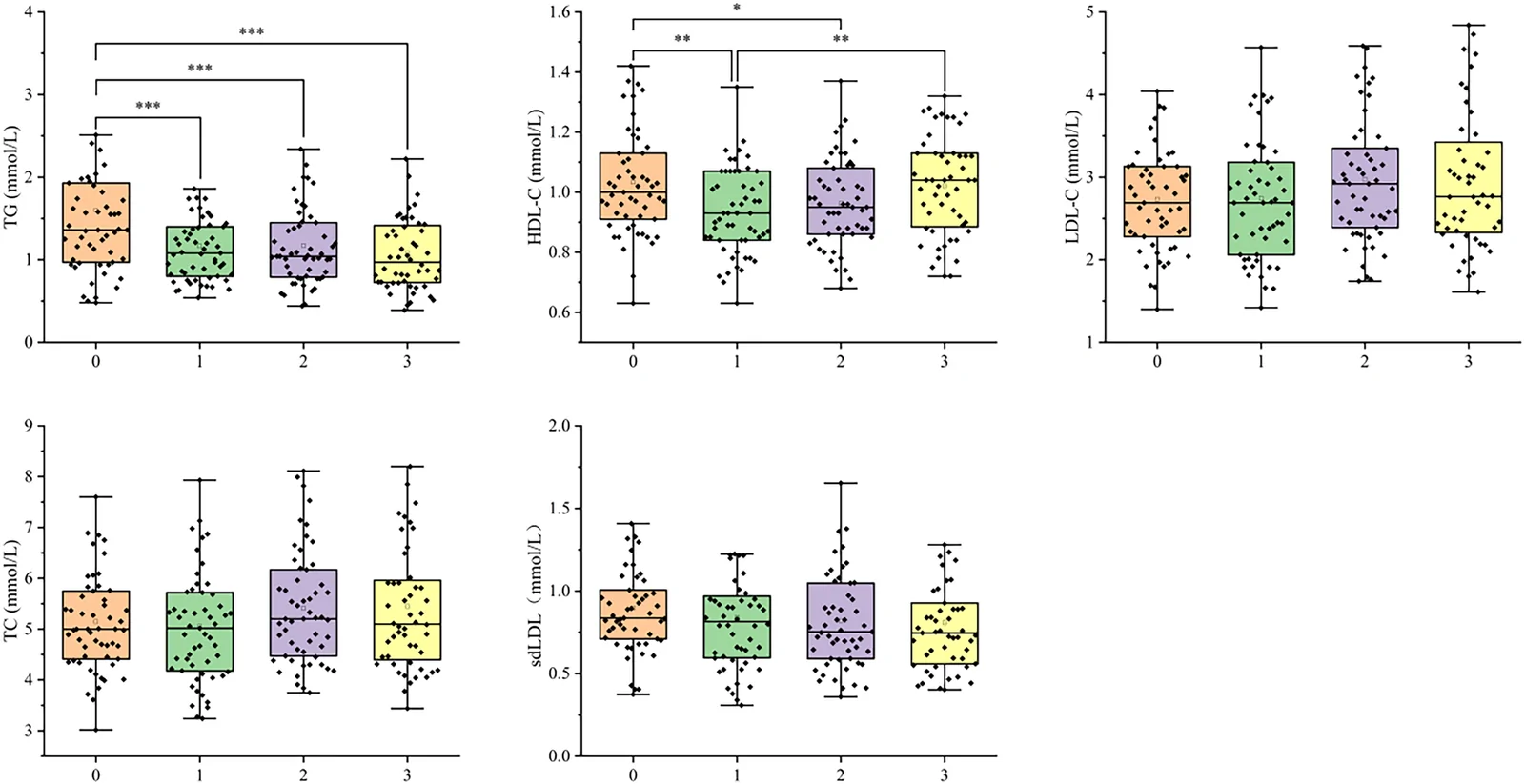
Changes in the lipid profile during the Eastern Healthy Dietary Pattern-modified ketogenic diet intervention. TG, triglycerides; HDL-C, high-density lipoprotein cholesterol; LDL-C, low-density lipoprotein cholesterol; TC, total cholesterol; sd-LDL, small dense low-density lipoprotein cholesterol. The numeric x-axis codes denote assessment time points: 0, baseline (week 0); 1, week 4; 2, week 8; and 3, week 12. Brackets identify the compared time points. ***P < 0.001; **P < 0.01; *P < 0.05.

Liver enzymes and ultrasonographic liver measures also improved (**Figure 4**). ALT decreased by 34.7%, from 38.52 ± 15.68 to 25.17 ± 10.34 U/L, and AST decreased by 28.8%, from 32.15 ± 12.47 to 22.89 ± 9.65 U/L (both *P* < 0.001). Liver stiffness decreased from 5.8 ± 1.3 to 4.9 ± 1.1 kPa (*P* < 0.01), and the liver attenuation coefficient decreased from 274.5 ± 35.8 to 241.2 ± 28.6 dB/m (*P* < 0.001). These changes paralleled the reduction in VFA and suggest improvement in hepatic steatosis and visceral adiposity.

**Figure 4:**
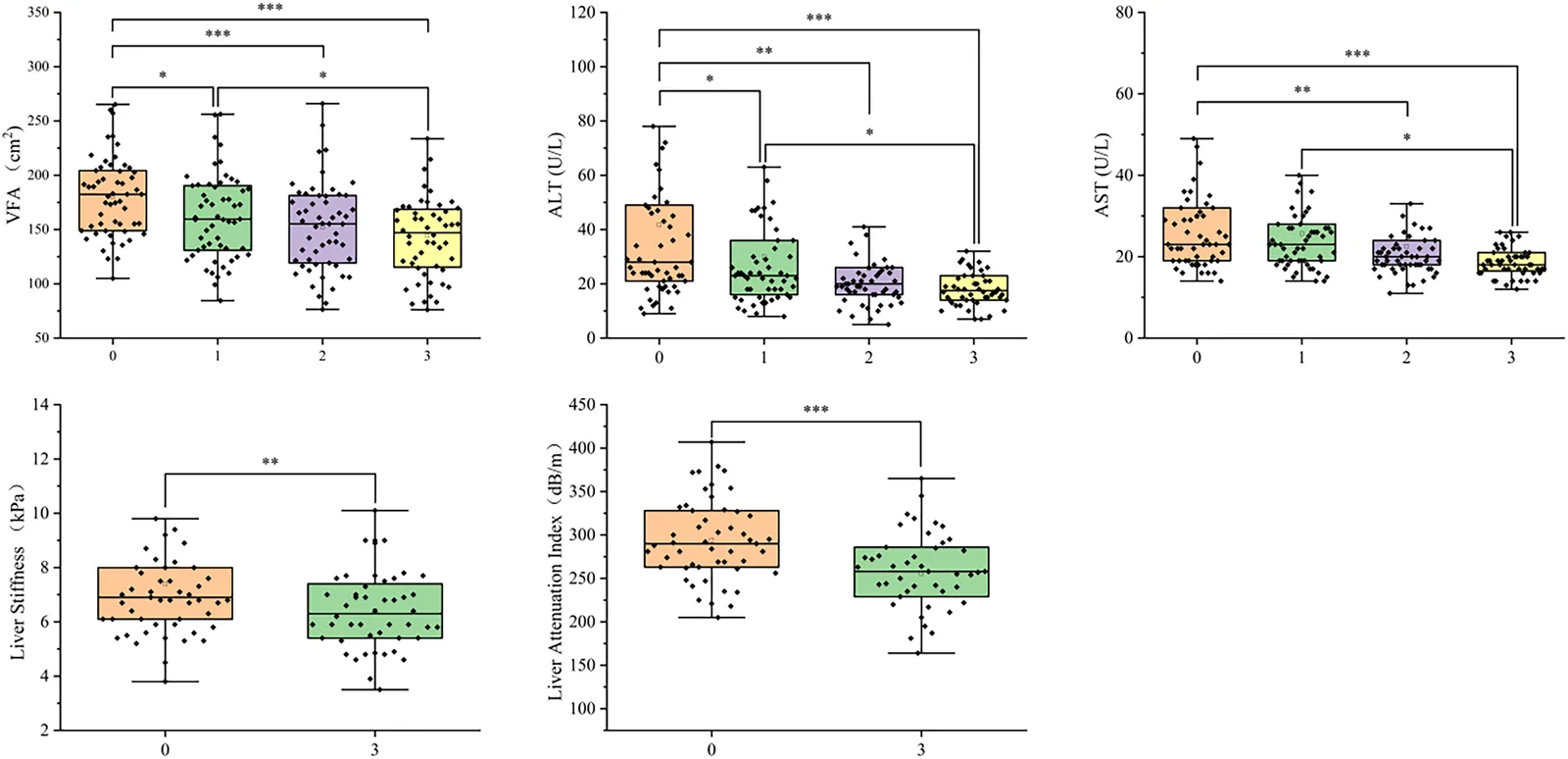
Changes in visceral fat area, liver enzymes, and ultrasonographic liver measures during the intervention. ALT, alanine aminotransferase; AST, aspartate aminotransferase. The numeric x-axis codes denote assessment time points: 0, baseline (week 0); 1, week 4; 2, week 8; and 3, week 12. Panels displaying only 0 and 3 were assessed only at baseline and week 12. Brackets identify the compared time points. ***P < 0.001; **P < 0.01; *P < 0.05.

### 3.3 Reproductive hormones and serum vitamin D

Reproductive hormone profiles and serum vitamin D changed after the 12-week intervention. Detailed results are presented in **Table 2**.

**Table 2:** Reproductive hormone and vitamin D concentrations at baseline and week 12.

Variable	Unit	Baseline	Week 12	*P* value
Follicle-stimulating hormone (FSH)	mIU/mL	5.46 ± 1.48	5.46 ± 1.37	0.481
Luteinizing hormone (LH)	mIU/mL	10.61 ± 5.21	4.72 (3.54, 6.28)	<0.001
Estradiol (E2)	pg/mL	209.90 (168.65, 281.90)	162.40 (137.55, 223.55)	0.005
Progesterone (PRO)	ng/mL	0.81 (0.56, 1.31)	1.04 (0.65, 1.44)	0.459
Total testosterone (T)	ng/dL	1.85 ± 0.62	1.34 ± 0.56	<0.001
Prolactin (PRL)	ng/mL	270.40 (199.80, 393.30)	308.20 (192.65, 433.45)	0.524
Anti-Müllerian hormone (AMH)	ng/mL	5.56 (3.20, 7.81)	4.81 (3.11, 6.80)	0.075
25-hydroxyvitamin D [25(OH)D]	ng/mL	17.86 (15.27, 20.84)	23.90 (21.16, 28.16)	<0.001

Data are presented as mean ± SD for normally distributed variables or median (interquartile range) for non-normally distributed variables. AMH, anti-Müllerian hormone; E2, estradiol; FSH, follicle-stimulating hormone; LH, luteinizing hormone; PRL, prolactin; PRO, progesterone; T, total testosterone.

LH decreased by 55.5%, whereas FSH remained unchanged (*P* = 0.481), resulting in a lower LH/FSH ratio. Total testosterone decreased by 35.8% (*P* < 0.001). Estradiol also decreased (*P* = 0.005), whereas prolactin did not change significantly (*P* = 0.524). AMH remained unchanged (*P* = 0.075), suggesting no detectable reduction in ovarian reserve over the 12-week intervention. Serum 25(OH)D increased from 17.86 ± 3.52 to 23.90 ± 4.12 ng/mL (*P* < 0.001).

### 3.4 Clinical symptoms and safety

#### 3.4.1 Menstrual symptoms

Menstrual symptoms improved after the 12-week intervention (**Figure 5**). Mean cycle length decreased from 51.0 ± 33.4 days at baseline to 38.2 ± 21.3 days at week 12 (*P* < 0.001). Only 6 participants (10.9%) had a normal cycle length of 28–35 days at baseline; this proportion increased after the intervention. The range of LH/FSH ratios narrowed from 0.46-5.18 at baseline to 0.43-2.80 after the intervention. At baseline, 6 participants (10.9%) reported moderate dysmenorrhea, 1 (1.8%) reported severe dysmenorrhea, and 38 (69.1%) reported no dysmenorrhea. At week 12, no participant reported moderate or severe dysmenorrhea, 2 (3.6%) reported mild dysmenorrhea, and 96.4% reported no dysmenorrhea (*P* < 0.001). The distribution of menstrual blood-loss scores also improved significantly (*P* < 0.05).

**Figure 5:**
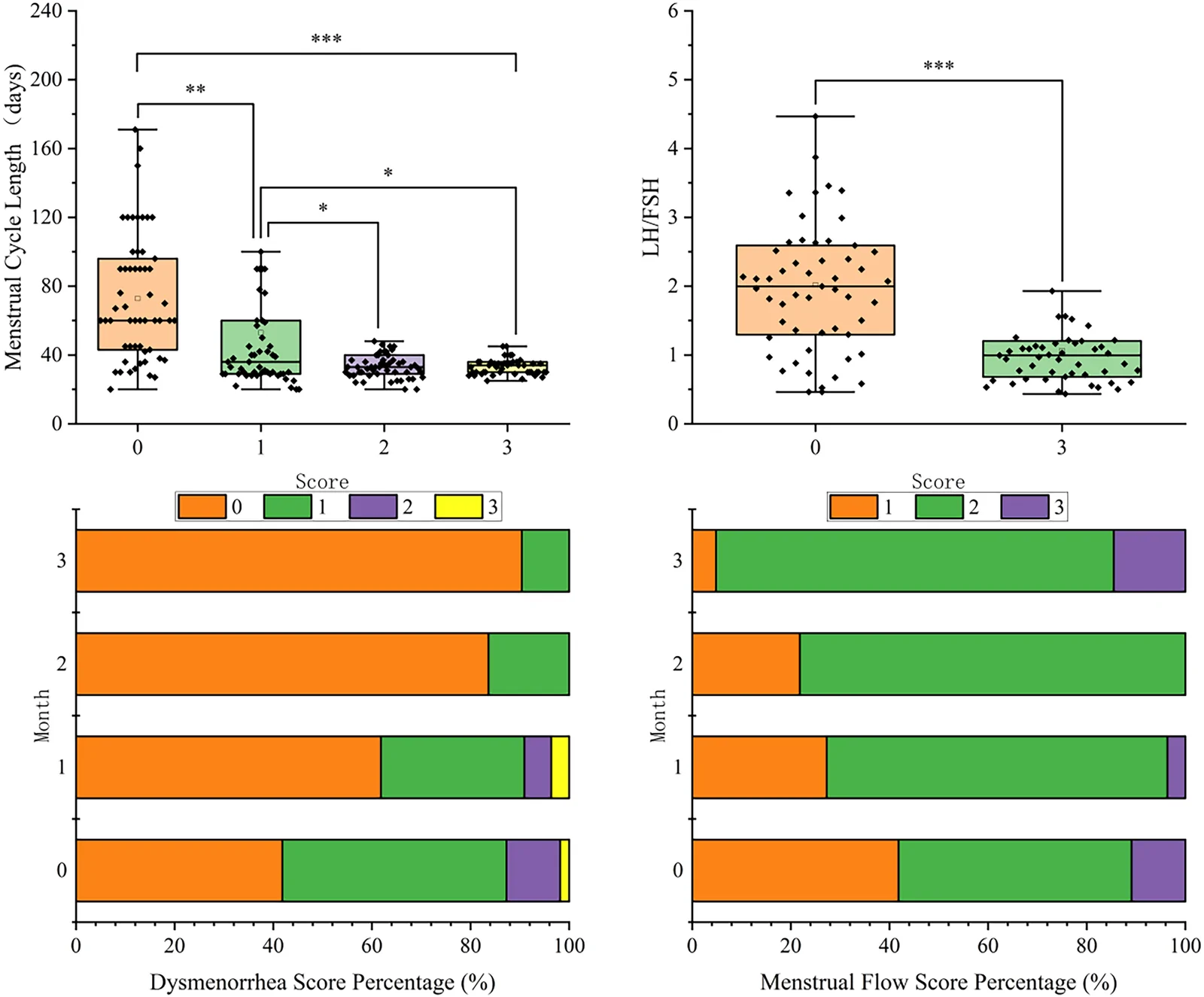
Changes in menstrual cycle length, the LH/FSH ratio, and menstrual symptom scores during the intervention. In the upper panels, numeric x-axis codes denote assessment time points: 0, baseline (week 0); 1, week 4; 2, week 8; and 3, week 12. In the stacked-bar panels, months 0–3 represent the same assessment time points, whereas colors represent symptom scores. Dysmenorrhea scores were defined as 0, no pain; 1, occasional mild pain without interference with daily activities; 2, frequent moderate pain causing mild interference; and 3, recurrent severe pain requiring medication and markedly limiting daily activities or requiring bed rest. Menstrual blood-loss scores were defined as 0, <80 mL; 1, 80–100 mL; 2, 100–120 mL; and 3, >120 mL. Brackets identify the compared time points. ***P < 0.001; **P < 0.01; *P < 0.05.

#### 3.4.2 Safety assessment

No clinically concerning changes were observed in the assessed safety parameters. White blood cell, neutrophil, lymphocyte, red blood cell, hemoglobin, and platelet values remained within the normal range and did not change significantly (all *P* > 0.05). BUN, Cr, and UA were also unchanged (all *P* > 0.05), indicating no detectable adverse effect on renal function. TP, ALB, TBIL, DBIL, and IBIL remained stable (all *P* > 0.05). Together with the improvements in ALT and AST, these findings indicate that the intervention was well tolerated over 12 weeks.

## 4 Discussion

### 4.1 Safety and nutritional features of the EHDP-modified ketogenic diet

A principal finding of this study was that the lipid profile improved despite fat providing 60%-70% of total energy: TG decreased by 32.5%, HDL-C increased modestly, and LDL-C and sd-LDL did not increase significantly. This pattern differs from concerns surrounding some conventional Western ketogenic diets, particularly those high in saturated fat (18). Several features of the EHDP-modified regimen may account for this finding. First, the regimen prioritized foods rich in unsaturated fatty acids, including camellia, olive, and flaxseed oils, nuts, fish, and shellfish, while restricting trans fats and excessive saturated fat. This composition may favorably influence cholesterol metabolism and reduce the risk of dyslipidemia (19). Second, the regimen targeted ≥25 g/day of dietary fiber from dark-green vegetables, edible mushrooms, konjac, and soy products; when food-based intake was insufficient, participants received 10–15 g/day of a fiber supplement. Fiber from both foods and supplements may have contributed to satiety, gastrointestinal tolerance, and the observed glycemic and lipid responses, while fish, shrimp, and soy products provided high-quality protein with relatively little saturated fat. Together, these components may reduce lipid absorption and support a favorable gut microbial environment (20). Third, the progressive induction of ketosis may have reduced gastrointestinal symptoms, hypoglycemia, and the metabolic stress associated with abrupt carbohydrate restriction, thereby supporting adherence.

Hematologic, renal, protein, and bilirubin indices remained stable throughout the intervention, and liver enzymes improved. These findings support the short-term tolerability of the EHDP-modified ketogenic diet and are consistent with the concept of flexible ketosis described in the Chinese expert consensus (11). Longer follow-up is required to determine whether this safety profile is maintained over time.

### 4.2 Innovation of the EHDP-modified ketogenic diet: comparison with other ketogenic approaches

The principal innovation of this study was the integration of the EHDP with a ketogenic diet to create a culturally adapted regimen for Chinese patients. Compared with a conventional Western ketogenic diet, the EHDP-modified regimen incorporates familiar foods typical of the Jiangnan diet, including fish, shrimp, soy products, and dark-green vegetables. This approach may improve palatability and long-term adherence. All 55 participants completed the 12-week intervention, and blood ketone concentrations remained within the target range, suggesting good short-term adherence (21). The target fiber intake of ≥25 g/day also addresses the low fiber content of many conventional ketogenic diets. Mediterranean-style ketogenic diets have shown favorable effects in PMOS (12), but some characteristic foods may be less familiar or accessible to patients in parts of China. By using locally available ingredients and simple cooking methods, the EHDP-modified regimen may be more readily integrated into customary Chinese dietary practices.

These findings provide preliminary evidence for a culturally adapted nutritional intervention for PMOS and support further evaluation of the EHDP-modified ketogenic diet in controlled clinical trials.

### 4.3 Clinical effects and potential mechanisms in patients with PMOS and obesity

Patients with PMOS and obesity in China have a high prevalence of abdominal obesity, insulin resistance, hyperandrogenism, menstrual irregularity, and infertility. The EHDP-modified ketogenic diet was designed to address these features. First, the intervention was associated with a 10.63% reduction in body weight, a 35.81 cm² reduction in VFA, and a 7.53 kg reduction in BFM, whereas SMM decreased by 1.60 kg. Preservation of muscle mass is clinically important because skeletal muscle contributes to insulin sensitivity and resting energy expenditure (22). Second, reductions in visceral adiposity were accompanied by a 48.8% decrease in HOMA-IR, a 35.8% decrease in total testosterone, and a lower LH/FSH ratio. These concurrent changes are consistent with attenuation of the obesity-insulin resistance-hyperandrogenism cycle, although causality cannot be established from this single-arm study. Third, reductions in liver enzymes, liver stiffness, and the attenuation coefficient suggest improvement in hepatic steatosis, which is clinically relevant because nonalcoholic fatty liver disease commonly coexists with PMOS (23). Finally, cycle length, dysmenorrhea, and menstrual blood-loss scores improved, and 96.4% of participants reported no dysmenorrhea at week 12. These clinical changes may reflect improvements in metabolic and reproductive endocrine function (24).

Although the reduction in fat mass exceeded the reductions in muscle-related measures, the significant decreases in SMM (1.60 kg) and FFM (2.44 kg) should not be minimized. The concomitant decrease in TBW may have influenced bioelectrical-impedance estimates of FFM, but preservation of lean tissue remains clinically important. The present dietary protocol emphasized adequate protein intake but did not include a structured resistance-exercise program. Future interventions should combine individualized protein distribution with progressive resistance exercise and monitor muscle strength and physical function to mitigate lean-mass loss.

Overall, the observed clinical effects may have been mediated in part by reductions in visceral adiposity and the accompanying improvements in insulin resistance and reproductive hormone profiles. Mechanistic studies are needed to confirm these pathways.

### 4.4 Menstrual symptom changes in relation to metabolic and endocrine responses

In this study population, menstrual changes occurred in parallel with marked metabolic and endocrine responses. Mean cycle length decreased from 51.0 to 38.2 days, moderate or severe dysmenorrhea was no longer reported at week 12, and menstrual blood-loss scores shifted toward lower categories. Over the same period, body weight and visceral fat area decreased, HOMA-IR fell by 48.8%, total testosterone decreased by 35.8%, and LH and the LH/FSH ratio declined. This pattern is biologically coherent with the endocrine-metabolic phenotype of PMOS: reduced adiposity and hyperinsulinemia may lessen ovarian androgen drive and support more regular hypothalamic-pituitary-ovarian signaling. Lifestyle intervention trials in women with obesity and the former PCOS diagnosis have likewise reported improved menstrual regularity (25).

The improvement in dysmenorrhea and estimated blood loss may also reflect more regular cycles and altered endometrial exposure; however, this interpretation remains exploratory. Ovulation was not confirmed, progesterone was not measured longitudinally, and participant-level correlations between changes in HOMA-IR, adiposity, reproductive hormones, and menstrual outcomes were not analyzed. Accordingly, the parallel changes observed here do not establish that the metabolic improvements mediated the menstrual response.

### 4.5 Limitations and future directions

This study has several limitations. First, it was a single-center, single-arm, self-controlled study with a relatively small sample of 55 participants and no parallel control group, such as a conventional ketogenic diet or the EHDP alone. These features limit causal inference and generalizability. Second, the intervention lasted only 12 weeks; therefore, its long-term effectiveness, adherence, and safety remain uncertain. Third, we did not assess potential mechanistic mediators such as the gut microbiota, inflammatory markers, or adipokines. Fourth, the study included only patients with obesity (BMI ≥28 kg/m²), and the findings may not apply to patients with overweight (BMI 24.0-27.9 kg/m²) or normal weight. Fifth, a 10–15 g/day fiber supplement was provided when food-based fiber intake was insufficient, but individual supplement exposure and its independent contribution were not quantified; thus, the effects of the food-based dietary pattern cannot be fully separated from those of supplemental fiber. Sixth, participant-level correlation or mediation analyses linking metabolic or endocrine changes to menstrual outcomes were not performed, so the observed parallel changes should not be interpreted as causal.

Additionally, no formal *a priori* sample-size calculation was performed; therefore, the modest sample may have limited statistical power and the precision of effect estimates.

Future studies should include adequately powered multicenter randomized controlled trials comparing the EHDP-modified ketogenic diet with a conventional ketogenic diet, the EHDP alone, and a standard energy-restricted diet. Longer follow-up is needed to assess durability, safety, adherence, and the optimal intervention duration. Multi-omics approaches, including metagenomics, metabolomics, and transcriptomics, may help clarify changes in the gut microbiota, inflammatory pathways, and adipokines. Future protocols should record supplement exposure quantitatively and prespecify correlation or mediation analyses of metabolic, endocrine, and menstrual outcomes. Studies should also include patients across BMI categories to inform individualized dietary recommendations.

## 5 Conclusion

In this single-arm, self-controlled study, a 12-week EHDP-modified ketogenic diet was associated with reductions in body weight, visceral adiposity, insulin resistance, triglycerides, liver-related indices, and androgen excess, together with improvements in menstrual symptoms. The regimen did not adversely affect the measured atherogenic lipid, hematologic, renal, protein, or bilirubin indices and was completed by all participants. By incorporating foods and cooking practices familiar to Chinese patients, this culturally adapted dietary approach may offer a practical nutritional option for patients with PMOS and obesity. Randomized controlled trials with larger samples and longer follow-up are needed to confirm its comparative effectiveness and long-term safety.

## Data Availability

The raw data supporting the conclusions of this article will be made available by the authors, without undue reservation.
